# Genetically conditioned interaction among microRNA‐155, alpha‐klotho, and intra‐renal RAS in male rats: Link to CKD progression

**DOI:** 10.14814/phy2.16172

**Published:** 2024-10-07

**Authors:** L. M. Harrison‐Bernard, L. Raij, R. X. Tian, E. A. Jaimes

**Affiliations:** ^1^ Department of Physiology Louisiana State University Health Sciences Center New Orleans Louisiana USA; ^2^ Katz Family Division of Nephrology University of Miami Miller School of Medicine Miami Florida USA; ^3^ South Florida Veterans Administration Foundation Miami Florida USA; ^4^ Renal Service Memorial Sloan Kettering Cancer Center and Weill Cornell Medical College New York New York USA

**Keywords:** alpha‐klotho, hypertension, miR‐155, plasmin, salt sensitivity

## Abstract

Incident chronic kidney disease (CKD) varies in populations with hypertension of similar severity. Proteinuria promotes CKD progression in part due to activation of plasminogen to plasmin in the podocytes, resulting in oxidative stress‐mediated injury. Additional mechanisms include deficiency of renal alpha‐klotho, that inhibits Wnt/beta‐catenin, an up regulator of intra‐renal renin angiotensin system (RAS) genes. Alpha‐klotho deficiency therefore results in upregulation of the intra‐renal RAS via Wnt/beta‐catenin. In hypertensive, Dahl salt sensitive (DS) and spontaneously hypertensive rats (SHR), we investigated renal and vascular injury, miR‐155, AT1R, alpha‐klotho, and TNF‐α. Hypertensive high salt DS (DS‐HS), but not SHR developed proteinuria, plasminuria, and glomerulosclerosis. Compared to DS low salt (DS‐LS), in hypertensive DS‐HS alpha‐klotho decreased 5‐fold in serum and 2.6‐fold in kidney, whereas serum mir‐155 decreased 3.3‐fold and AT1R increased 52% in kidney and 77% in aorta. AT1R, alpha‐klotho, and miR‐155 remained unchanged in prehypertensive and hypertensive SHR. TNF‐α increased by 3‐fold in serum and urine of DS‐HS rats. These studies unveiled in salt sensitive DS‐HS, but not in SHR, a genetically conditioned dysfunction of the intermolecular network integrated by alpha‐klotho, RAS, miR‐155, and TNF‐α that is at the helm of their end‐organ susceptibility while plasminuria may participate as a second hit.

## 1 INTRODUCTION

Chronic kidney disease (CKD) is a growing public health concern affecting nearly 26 million Americans that is associated with increased mortality mostly cardiovascular (CVD) (Saran et al., 2018). Hypertension is the second leading cause of end‐stage kidney disease (ESKD); however, the incidence of CKD varies in populations with HTN of similar severity (Eriksen et al., 2016; Hsu et al., 2003). The role of glomerular barotrauma associated with hypertension is well established (Cowley Jr., 2013; Wang et al., 2013), although barotrauma by itself does not fully explain hypertensive glomerular injury (Drawz et al., 2017; Feng et al., 2015, 2017; Hostetter & Rosenberg, 1990; Xie et al., 2016). The prevalence of salt sensitivity is increased in blacks that confers an increased risk for the development of hypertension, cardiovascular and renal disease. Indeed in the US, the incidence of ESRD among blacks is three‐fold higher than that of whites (System, 2020), despite a similar prevalence of CKD. Moreover, the recent SPRINT trial (Drawz et al., 2017), as well as, meta‐analysis of 19 trials (Xie et al., 2016), have shown that intensive versus standard systolic blood pressure control offers a survival benefit, but does not arrest CKD progression and long term risk of ESRD (Langefeld et al., 2015), particularly when proteinuric patients reach eGFR 30–45 (stage 3–4) (Ku et al., 2018). Furthermore, the remarkable CVD risk of CKD patients (Coresh et al., 2007; Foster et al., 2013) has not been fully elucidated by traditional risk factors, suggesting the participation of “CKD specific risk factors” (Sarnak et al., 2003) for which current preventative therapies remain insufficient.

Alpha‐klotho is a molecule with beneficial pleiotropic renal and extra renal effects (Hu et al., 2013). While there is a physiological slight reduction in alpha‐klotho with aging (Drew et al., 2017; Kuro‐o et al., 1997; Kurosu et al., 2005), more pronounced reductions in renal and circulating alpha‐klotho are one of the earliest manifestations of CKD (Shi et al., 2016). Physiologically, alpha‐klotho represses Wnt/b –catenin, the leading upstream regulator of multiple intra‐renal RAAS genes (Floege, 2015). Thus, CKD can be characterized as a “state of intra‐renal RAAS upregulation” linked to the concurrent deficiency of alpha‐klotho and upregulation the Wnt/b–catenin pathway (Zhou et al., 2013; Zhou, Li, et al., 2015; Zhou, Mo, et al., 2015).

MicroRNAs (miRNAs) are central epigenetic regulators that take part in RNA silencing and posttranscriptional regulation of gene expression (Small & Olson, 2011).

Assessment of available clinical and preclinical data (Chen et al., 2013; DuPont et al., 2016; Hu et al., 2013, 2017; Zhou et al., 2013) led us to hypothesize that miR‐155 interaction with alpha‐klotho, RAAS, and the pro‐inflammatory tumor necrosis factor alpha (TNF‐alpha) might be a previously unsuspected link in the development and progression of CKD and CVD. Previous studies demonstrated that glomerular injury in Dalh salt sensitive rats (DS‐HS) is linked to podocyte injury. Podocytes are critical for maintaining the glomerular “filter integrity” and proteinuria associated with podocyte loss is a forerunner of CKD progression (Raij et al., 2016; Wickman et al., 2013). We have shown that plasmin, present in nephrotic urine of humans and rodents (plasminuria), binds to podocytes, activates the production of reactive oxidative species and promotes disruption of the podocyte's cytoskeletal architecture and death (Hinrichs et al., 2018; Raij et al., 2016; Wickman et al., 2013). We hypothesize that plasminuria partakes as a “second hit” that fosters further podocyte/glomerular injury (Ku et al., 2018; Raij et al., 2016; Wickman et al., 2013). To test the above hypothesis, we performed parallel studies in age‐matched, similarly hypertensive high salt Dahl salt sensitive (DS‐HS) and spontaneously hypertensive rats (SHR); two genetic models of hypertension that mirror important aspects of renal injury found in human essential hypertension as well as in vitro studies in podocytes (Cowley Jr., 2013; Raij, 2003; Raij et al., 1984).

## 2 METHODS

### 2.1 Animal protocols

All rats were housed in facilities accredited by the American Association for Accreditation of Laboratory Animal Care at the Miami VAMC. The Institutional Animal Care and Use Committee at the Miami VA Medical Center approved the studies. SHR rats were purchased from Charles River (Germantown, NY). Male DS and Dahl salt‐resistant (DR) rats were purchased from Harlan Sprague–Dawley (Indianapolis, IN) and maintained under controlled conditions of light, temperature, and humidity. After 2‐week accommodation to the new environment, the rats were divided into groups. SHR were used for experiments at 6 (*n* = 8), 16 (*n* = 8), and 52 (*n* = 4) weeks of age. SHR rats were fed standard rat chow that contained 0.5% NaCl. Six‐week‐old DS and DR rats were fed standard rat chow that contained either 4% NaCl (DS‐HS: *n* = 5 and DR‐HS: *n* = 4) or 0.5% NaCl (DS‐LS: *n* = 8 and DR‐LS: *n* = 8) for 8 weeks. DS and DR groups were used for the experiments at the age of 16 weeks. All rats had free access to water. Systolic blood pressure was measured in conscious rats by the tail‐cuff method. Twenty‐four‐hour urinary excretion was collected in individual metabolic cages. At the end of the study, the rats were euthanized by decapitation, and blood, heart, kidneys, and aorta were harvested, weighed, and frozen in liquid nitrogen and stored at −80°C until use. Kidney samples were fixed and embedded in paraffin, sectioned, and stained with Periodic acid‐Schiff. Left ventricle (LV) weight and aortic wet weight/cm (arch of thoracic aorta to the origin of mesenteric artery) were used as indices of left ventricular hypertrophy (LVH) and aortic hypertrophy, respectively.

### 2.2 In vitro studies

Male human podocytes initially generated by MA Saleem Children's Renal Unit and Academic Renal Unit, University of Bristol, UK (Wang et al., 2012) were generously provided by S. Merscher‐Gomez and A. Fornoni, University of Miami. Briefly, human podocytes were cultured and differentiated in RPMI 1640 culture medium containing 10% FBS, 1% penicillin/streptomycin with or without 1% ITS, 50 U/mL human interferon as described (Ruiz‐Andres et al., 2016; Wang et al., 2012). The immortalized normal human podocytes were propagated at 33°C and then thermoshifted for differentiation for 14 days at 37°C. Terminally differentiated podocytes were starved in serum‐free RPMI 1640 medium for 24 h before the experiments were performed. Human podocytes were treated with plasminogen (Plg, 1 μmoL/l), the concentration of Plg found in human serum (Ma et al., 2016), for 6 h. The cells were preincubated with ML204, TRPC4/C5 ion channel antagonist (Sigma Aldrich, 30 μmoL/l), for 30 min before incubation with Plg. The cells were harvested with lysis buffer containing a cocktail of protein inhibitor (Complete Mini, Roche Diagnostics GmbH, cat. no.11836153001). Proteins were quantified by Bio‐Rad assay, and 30 mg of total protein were first subjected to SDS‐PAGE and then transferred to nitrocellulose membranes. The membranes were incubated overnight in a cold room with primary rabbit anti‐CD36 (Santa Cruz SC‐9154), anti‐talin (Cell Signaling Technology #4021), anti‐Nrf2 (Santa Cruz SC‐722), anti‐CD151 (Santa Cruz Biotechnology SC‐33123), followed by incubation with a peroxidase‐conjugated secondary antibody for 1 h.

### 2.3 Western blots

Proteins were quantified by Bio‐Rad assay, and 30 mg of total protein were first subjected to SDS‐PAGE and then transferred to nitrocellulose membranes. The membranes were incubated overnight in a cold room with one of the following antibodies anti‐pJAK2 (Cell Signaling #8080), anti‐tubulin (Santa Cruz: SC‐9104), anti‐pMYPT1 (Santa Cruz SC‐17556R1), anti‐AT1R (Santa Cruz SC‐57036), anti‐ Arhgef1 (Santa Cruz SC‐20804), anti‐CD36 (Santa Cruz SC‐9154), anti‐talin (Cell Signaling Technology #4021), anti‐Nrf2(Santa Cruz SC‐722), anti‐CD151 (Santa Cruz SC‐33123), followed by incubation with a peroxidase‐conjugated secondary antibody for 1 h. All the Ab used equivalence‐of‐protein loading and transfer was confirmed by reblotting the samples with anti‐beta‐actin antibody. Immune reactive bands were detected by chemiluminescence and quantified by densitometry. Relative quantities of each protein were normalized by beta‐actin (Santa Cruz, SC69879) and expressed as fold increase versus control. All the Ab used equivalence‐of‐protein loading and transfer was confirmed by reblotting the samples with anti‐beta‐actin antibody. Immune reactive bands were detected by chemiluminescence and quantified by densitometry. Relative quantities of each protein were normalized by beta‐actin (Santa Cruz, SC69879) and expressed as fold increase versus control.

### 2.4 RT‐PCR

Total RNA was extracted from normal veins and AVFs with TRIzol (Invitrogen, Carlsbad, CA), treated with DNase I and then purified with an RNA purification kit (Invitrogen, Carlsbad, CA). The protein‐ and DNA‐free RNA was reverse‐transcribed to cDNA (Invitrogen, Carlsbad, CA) and amplified by PCR with specific primers and quantified using SYBR Green and a 7300 Real Time PCR System (Applied Biosystems, Foster City, CA) as previously described (Rezonzew et al., 2012). Levels of specific mRNAs were normalized using glyceraldehyde 3‐phosphate dehydrogenase as an internal control.

The following Klotho primers were used (Applied Biosystems, Foster City, CA # 4351372) and miR‐155 sense primer: 5‐GGAGGTTAATGCTAATTGTGATAG‐3; miR‐155 antisense primer: 5‐GTGCAGGGTCCGAGGT‐3, from Genomic Life, La Jolla, CA, Rn0676072m1.

### 2.5 Plasminogen, klotho and TNF‐

Urinary plasminogen (ICLAB E‐25PMG), serum Klotho (Cusabio Technology, Houston TX, CSB‐E14958R) and TNF‐**α** (R&D Life Biosciences, Minneapolis, MN, RTA00) were measured by Elisa following the manufactures instructions and adjusted for urinary creatinine.

### 2.6 Kidney injury

Glomerular injury score (GIS) was measured in PAS stained kidney sections as previously described (Raij et al., 1984). A minimum of 20 glomeruli (range, 20 to 60) in each specimen was examined and the severity of the lesion was graded from 0 to 4 + according to the percentage of glomerular involvement. Thus, a 1 + lesion represented an involvement of 25% of the glomerulus while a 4+ lesion indicated that 100% of the glomerulus was involved. An injury score was then obtained by multiplying the degree of damage (0 to 4+) by the percentage of the glomeruli with the same degree of injury. The extent of the injury for each individual tissue specimen was then obtained by the addition of these scores.

### 2.7 Statistical analysis

Results were expressed as means ± SD. The data were evaluated by 1‐way or 2‐way ANOVA. When the overall *F* test result of ANOVA was significant, a multiple‐comparison Dunnett test was applied. Student *t* test was used in 2 mean comparisons. Differences were reported as significant when *p* value was <0.05.

## 3 RESULTS

### 3.1 Hypertension and proteinuria

**FIGURE 1:**
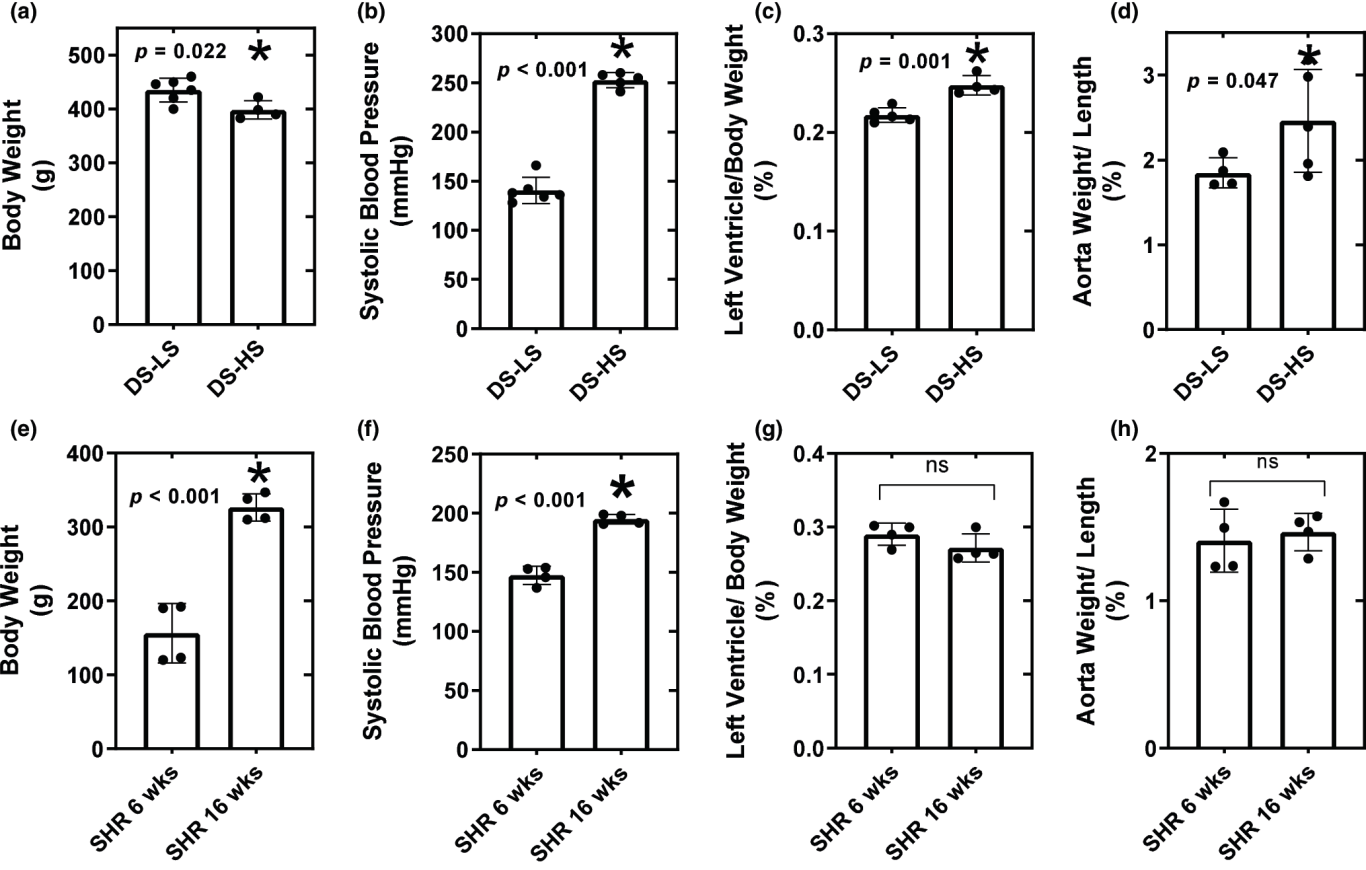
Body weight; (a), systolic blood pressure; (b), left ventricle/body weight ratio; (c), and aorta weight/length; (d) for DS‐LS (*n* = 6) and DS‐HS (*n* = 5) rats. *p ≤ 0.05 versus DS‐LS. Body weight; (e), systolic blood pressure; (f), left ventricle/body weight ratio (g), and aorta weight/length (h) for SHR 6 week (*n* = 8) and SHR 16 week (*n* = 8). *p ≤ 0.05 versus SHR 6 week.

DS‐HS rats had significantly lower body weight (BW) as compared to DS‐LS rats (Figure 1a). SBP, LV/BW ratio, and aorta weight/length were significantly elevated in DS‐HS compared to DS‐LS rats (Figure 1b–d). SHR at 16 weeks of age exhibited significantly elevated BW and SBP compared to SHR at 6 weeks of age (Figure 1e,f). LV/BW and aorta weight/length were not different between the 6 and 16‐week‐old SHR (Figure 1g,h). Urinary protein excretion averaged 89.1 ± 1.7 and 27.0 ± 3.4 mg/day (*p* < 0.05) in DS‐HS and DS‐LS rats, respectively. In contrast, urinary protein excretion averaged 7.8 ± 1.0, 9.4 ± 0.9, and 11.9 ± 2.2 mg/day in 6, 16, and 52‐week‐old SHR, respectively (P=NS).

### 3.2 Aortic AT1R, p‐JAK/JAK2, p‐MYPT1/MYPT1, and Arhgef1

**FIGURE 2:**
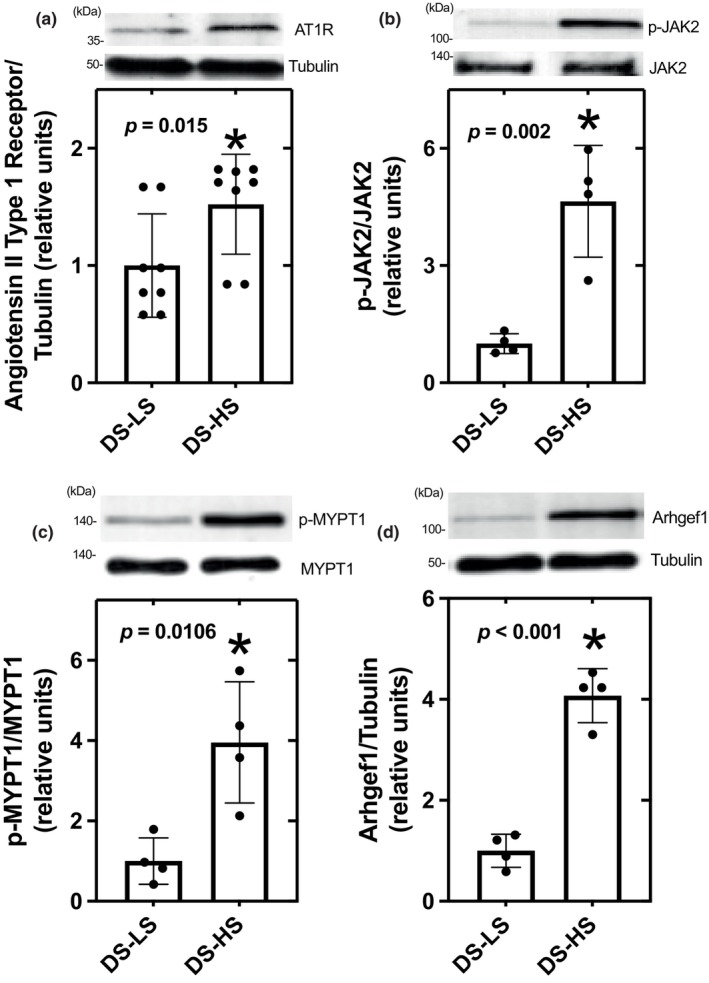
AT1R; (a), p‐JAK2; (b), p‐MYPT1; (c), and Arhgef1; (d) protein expression in aorta of DS‐LS and DS‐HS rats, *p ≤ 0.05 versus DS‐LS.

**FIGURE 3:**
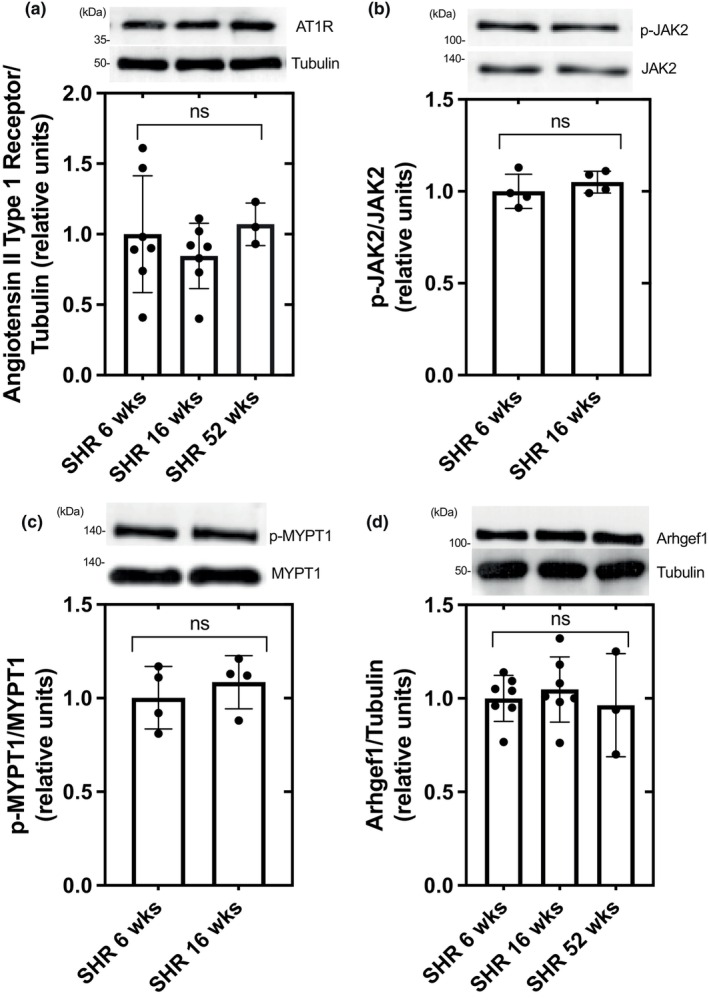
AT1R (a), p‐JAK2 (b), p‐MYPT1 (c), and Arhgef1 (d) protein expression in aorta of SHR 6 week (*n* = 8) and SHR 16 week (*n* = 8).

Aortic AT1R, p‐JAK/JAK2, p‐MYPT1/MYPT1, and Arhgef1 protein levels were significantly elevated in DS‐HS compared to DS‐LS rats (Figure 2a–d), while there were no differences in these protein levels in 6‐week‐old compared to 16‐week‐old SHR (Figure 3a–d). Aortic AT1R and Arhgef1 protein expression was not different between 52‐week‐old and 6‐week‐old SHR (Figure 3a,d). HS diet did not alter p‐JAK/JAK2 protein levels in DR rats as compared to LS diet (1.02 ± 0.12 vs. 1.00 ± 0.13).

### 3.3 TNF **‐α** and urinary plasminogen

**FIGURE 4:**
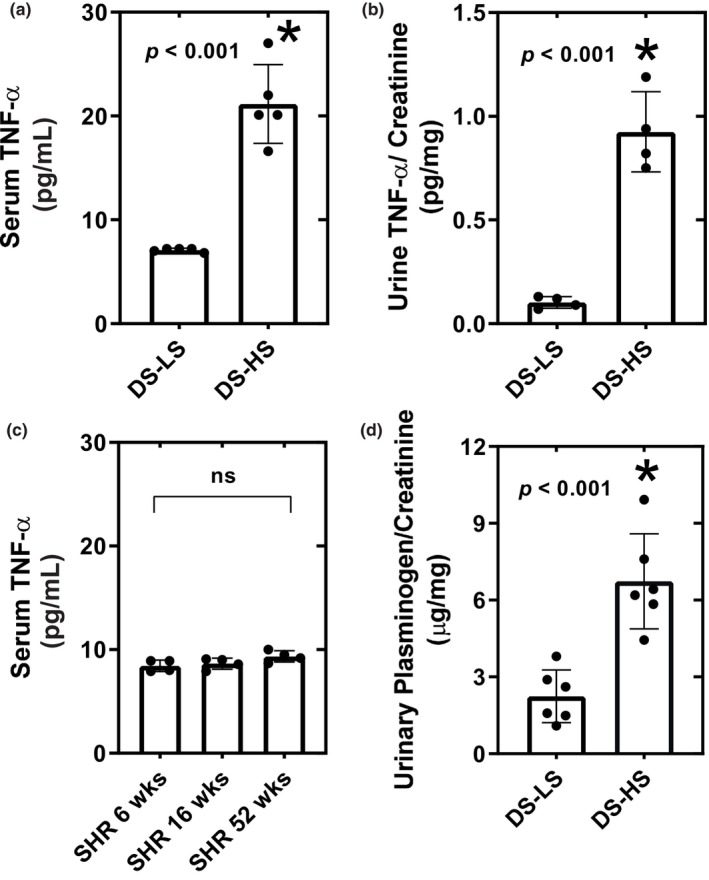
Serum TNF‐alpha (a) and urine TNF‐alpha/creatinine (b) for DS‐LS (*n* = 5) and DS‐HS (*n* = 5) rats. vs. DS‐LS. Serum TNF‐alpha (c) for SHR 6 (*n* = 5), SHR 16 (*n* = 5), and SHR 52 week (*n* = 5) (d) Urinary plasminogen for DS‐LS (*n* = 6) and DS‐HS (*n* = 6).

Compared to DS‐LS rats, serum (Figure 4a) and urine (Figure 4b) TNF‐**α** levels were elevated 3‐fold in DS‐HS rats. In contrast serum TNF‐**α** levels were not different between 6‐week, 16‐week, and 52‐week‐old SHR rats (Figure 4c). In addition, urinary plasminogen excretion was significantly elevated in DS‐HS compared to DS‐LS rats (Figure 4d).

### 3.4 Renal injury

**FIGURE 5:**
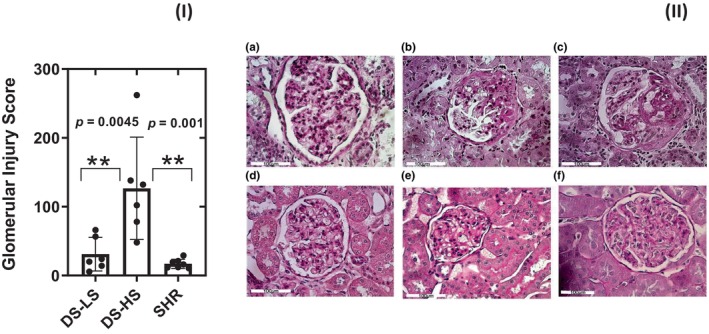
(I) Bar graph showing glomerular injury score for DS‐LS, DS‐HS and SHR rats at 52 weeks. (II) Representative Periodic acid‐Schiff staining of glomeruli from DS‐LS (a), DS‐HS (b, c) and 6 (d), 16 (e), and 52 (f) week old SHR. Only kidneys of DS‐HS rats demonstrate glomerulosclerosis.

**FIGURE 6:**
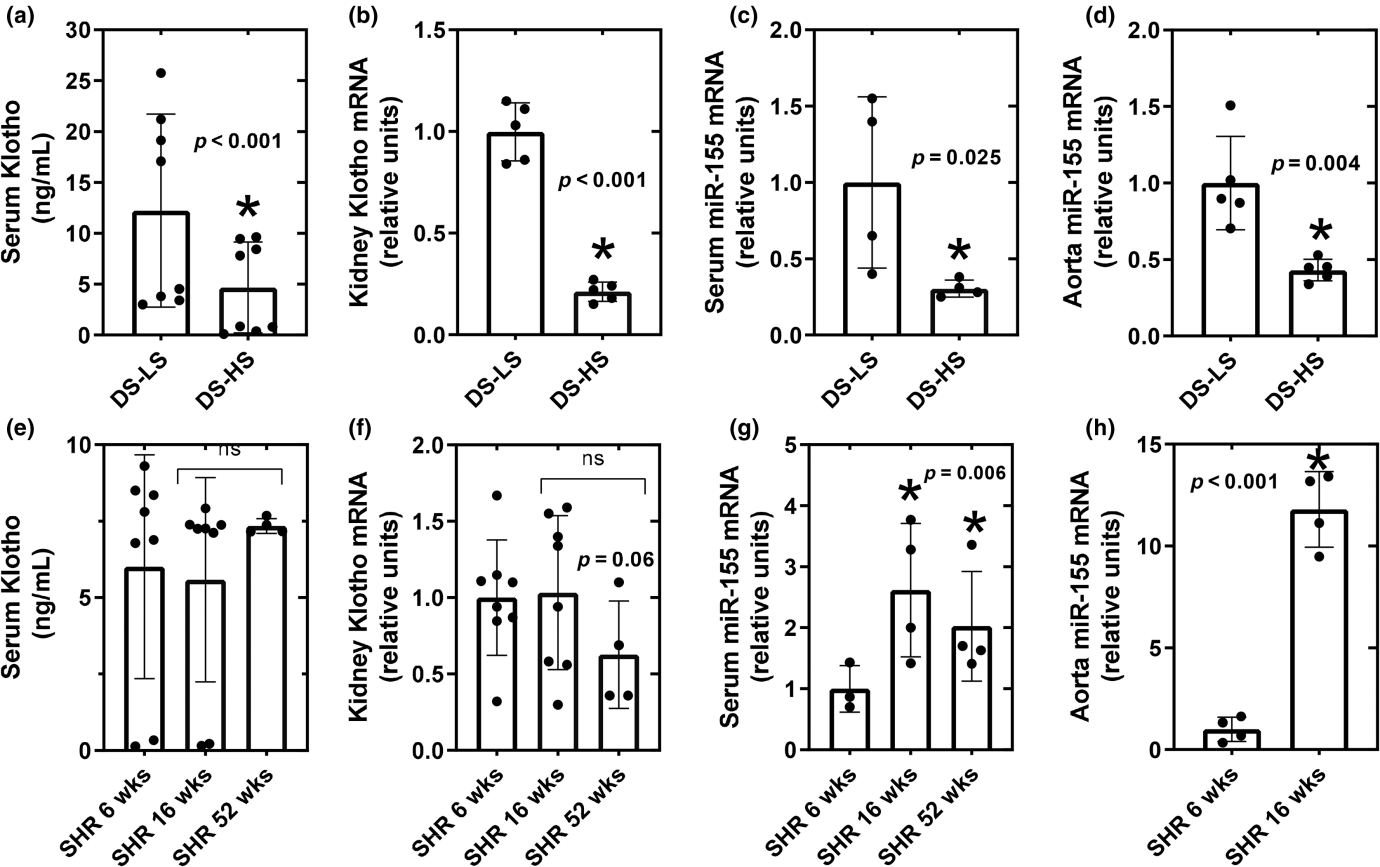
Serum alpha‐klotho; (a), and kidney alpha‐klotho; (b), serum miR‐155; (c), and aorta miR‐155; (d) mRNA expression in DS‐LS and DS‐HS rats. *p ≤ 0.05 versus DS‐LS. Serum alpha‐klotho (e), and kidney alpha‐klotho (f), serum miR‐155; (g), and aorta miR‐155; (h) mRNA expression in SHR‐6 weeks (*n* = 8) and SHR‐16 weeks (*n* = 8). *p ≤ 0.05 versus SHR‐6 weeks.

Quantification of the glomerular injury score showed that only DS‐HS had evidence of glomerulosclerosis (Figure 5 bar graph). Photomicrographs are representative of PAS staining of glomeruli from DS‐LS (Figure 5a), DS‐HS (Figure 5b,c) and 6 (Figure 5d), 16 (Figure 5e), and 52 (Figure 5f) week‐old SHR rats.

### 3.5 Klotho

Serum klotho concentrations and kidney klotho mRNA levels were significantly lower in DS‐HS compared to DS‐LS rats (Figure 6a,b); however, the levels were not different between hypertensive 16‐week‐old SHR and prehypertensive SHR rats (Figure 6e,f), nor were the levels different compared to 52‐week‐old SHR rats (7.35 ± 0.12 ng/mL; 0.66 ± 0.19 relative to 6‐week SHR), respectively.

### 3.6 Aortic and serum miR‐155 mRNA

Serum miR‐155 mRNA levels in DS‐HS rats were significantly suppressed to 0.3‐fold compared to DS‐LS rats (Figure 6c). In addition, aorta miR‐155 mRNA levels in DS‐HS rats were significantly suppressed to 0.4‐fold compared to DS‐LS rats (Figure 6d). Serum miR‐155 mRNA levels were significantly elevated by 2.6‐fold in 16‐week‐old compared to 6‐week‐old SHR rats (Figure 6g) and were still significantly elevated in 52‐week‐old SHR (2.33 ± 0.52‐fold compared to 6‐week‐old SHR rats). Aorta miR‐155 mRNA levels were significantly elevated by 12‐fold in 16‐week‐old compared to 6‐week‐old SHR rats (Figure 6h). HS diet did not alter aorta miR‐155 mRNA levels in DR rats compared to LS diet (1.02 ± 0.35 vs. 1.00 ± 0.24).

### 3.7 Kidney AT1R expression

**FIGURE 7:**
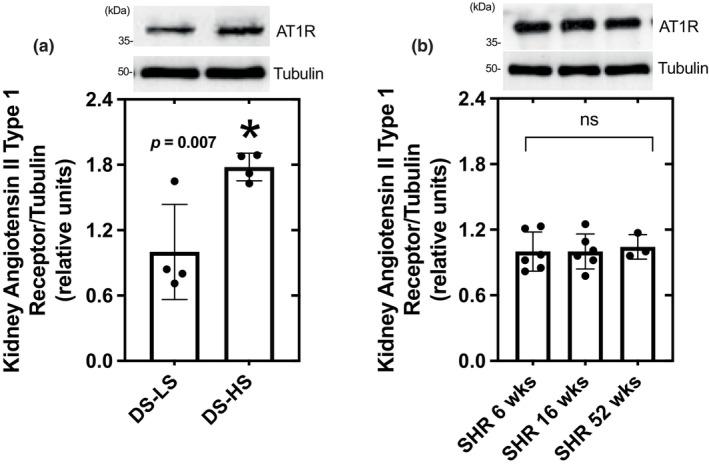
AT1R protein expression in kidney of DS‐LS and DS‐HS rats (a) and 6 week and 16 week SHR (b). vs. DS‐LS.

Kidney AT1R protein levels were significantly elevated in DS‐HS compared to DS‐LS (Figure 7a) rats. However, AT1R protein levels were not different between 6 week and 16‐week SHR rats (Figure 7b). Similar expression levels of the AT1R were found in kidneys of 52‐week SHR rats (1.05 ± 0.06 relative to 6‐week SHR).

### 3.8 In vitro studies

**FIGURE 8:**
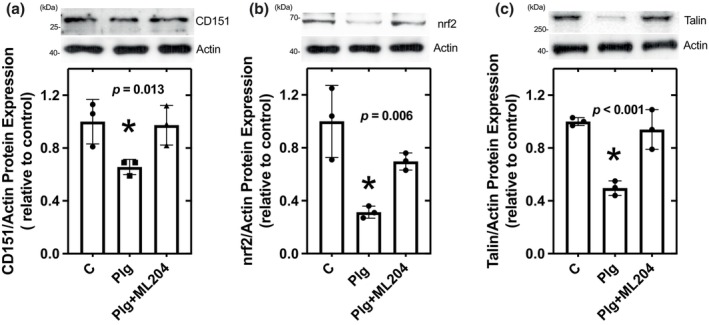
The effects of plasminogen plus/minus ML204 on CD151; (a), nrf2; (b), and Talin; (c) protein expression in cultured human podocytes.

Human podocytes incubated with plasminogen had a significant reduction in the protein expression of CD151 (Figure 8a), Nrf2 (Figure 8b), and Talin (Figure 8c). These effects of plasminogen were blocked by incubation with the potent, selective TRPC4/C5 and TRPC6 inhibitor, ML204 (Figure 8).

## 4 DISCUSSION

These studies were designed to identify new targets and pathways that could facilitate the development of mechanistically based new therapies to mitigate CKD progression. The novel findings of these studies are the unraveling of a dysfunctional, genetically conditioned molecular circuit, integrated by miR‐155, alpha‐klotho, the intra‐renal RAS, and TNF‐α that is linked to the development and progression of CKD.

**FIGURE 9:**
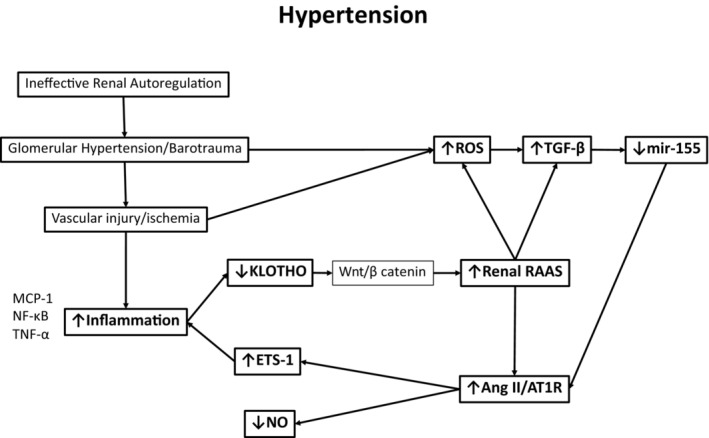
Mechanisms that participate in the development of vascular and renal injury in hypertension.

For these studies, we have resorted to comparative studies of two genetic models of hypertension that recapitulate many features of hypertension observed in humans particularly those prone to develop proteinuria and CKD progression and those that do not (Cowley Jr., 2013; Hayakawa & Raij, 1998; Shibata et al., 2011). Here we corroborated prior studies by us (Feng et al., 2015, 2017; Hayakawa et al., 1997; Raij et al., 1984, 1985) and others (Cowley Jr., 2013; Wang et al., 2013) that hypertensive DS‐HS, but not SHR rats developed severe proteinuria and widespread glomerular sclerosis. The glomerular injury of hypertensive DS‐HS rats is linked to barotrauma associated with glomerular hypertension and concurrent glomerular upregulation of the pro‐inflammatory transcription factor ETS‐1 (Avian Erythroblastosis Virus E26OncogenHomolog‐1) as we have previously demonstrated (Cowley Jr., 2013; Feng et al., 2015, 2017) (Figure 9).

Ang II binding to AT1R results in sequential phosphorylation/activation of JAK2 (pJAK2) and the GEF (guanine exchange factor), Arhgef1 (p‐Arhgef1) resulting in activation of a RhoA cascade known to be linked to activation of the calcium channel TRPC6 (transient receptor potential channel canonical‐6), as well as, activation of myosin light chain that promote vascular contraction, hypertrophy, and inflammation (Bernstein & Fuchs, 2010; Guilluy et al., 2010). Here we documented expression of p‐JAK2 and p‐Arhgef in aortas of DS‐HS rats, but not in similarly hypertensive age‐matched SHR; mechanistically, confirming in vivo vascular and renal activation of AT1R only in hypertensive DS‐HS, but not SHR rats (Arendshorst, 1997; Zhou et al., 2003).

MicroRNAs are small, noncoding RNAs that control gene/protein expression through target messenger RNA degradation and/or inhibition of protein synthesis. Previous studies have shown that miR‐155 is a powerful down‐regulator of AT1R (Chen et al., 2013; DuPont et al., 2016). Here we show that compared to 16‐week‐old nonhypertensive DS‐LS, 16‐week‐old hypertensive DS‐HS manifest striking miR‐155 reduction in serum (3.3‐fold) and aorta (2.3‐fold) accompanied by parallel upregulation of AT1R in aorta (52%) and kidney (78%). In remarkable contrast, similarly hypertensive SHR show during transition from 6 to 16 weeks of age a marked increase in serum (2.6‐fold) and aorta (12‐fold) miR‐155, and no kidney or aorta upregulation of AT1R.

Our novel findings showing selective miR‐155 down‐regulation in DS‐HS, but not in SHR brought up additional clinically, scientifically relevant questions regarding whether down‐regulation of miR‐155 is an aberrant response to hemodynamic and/ or inflammatory factors that only occurs or is more severe in genetically susceptible CKD subjects and whether is linked with the alpha‐klotho deficiency observed clinically and experimentally in CKD (Hu et al., 2013).

Alpha‐klotho, synthesized in the kidney, has anti‐senescence and pleiotropic renal and extra‐renal cardiovascular protective mechanisms (Hu et al., 2013, 2017). Wnt/beta catenin is a master upstream regulator that controls the intrarenal expression of multiple RAS genes including angiotensinogen, ACE, renin and AT1R (Zhou et al., 2013; Zhou, Li, et al., 2015; Zhou, Mo, et al., 2015). In the normal kidney, Wnt/beta catenin is maintained inactive by alpha‐klotho (Zhou et al., 2013). Loss of renal alpha‐klotho expression de‐represses Wnt/b catenin signaling followed by renal up‐regulation of angiotensinogen, renin, and AT1R protein levels (Figure 9) (Zhou, Li, et al., 2015; Zhou, Mo, et al., 2015). Here we show that serum alpha‐klotho concentrations and kidney alpha‐klotho mRNA expression were significantly lower in DS‐HS compared to DS‐LS rats, 2.6 and 5‐fold, respectively; on the other hand, alpha‐klotho levels were not different between hypertensive 16‐week‐old SHR and prehypertensive 6 week old SHR rats. TNFα is a major pro‐inflammatory cytokine involved in CKD progression (Ruiz‐Andres et al., 2016). In the current studies, we determined that although in hypertensive DS‐HS rats, serum and urine levels of TNFα are significantly increased, they are not increased in hypertensive SHR. Of note, TNFα has been shown to reduce renal alpha‐klotho expression via canonical NF‐kB pathway activation (Moreno et al., 2011; Zhou et al., 2013).

Podocytes are critical for maintaining the glomerular “filter integrity”, but are susceptible to damage and are limited in undergoing sufficient replication to compensate for podocyte loss once it exceeds 30%–40% (Wickman et al., 2013). Podocyte survival depends upon a balanced multimolecular interaction that includes Ang ll via the AT1R (Hoffmann et al., 2004; Raij et al., 2016) and the Rho GTpase Rac1 pathway (Wang et al., 2012; Zhou et al., 2017). A genetic (Wang et al., 2012) or disease related (Zhou et al., 2017) dysregulation of these pathways results in aberrant overproduction of ROS (Ruiz‐Andres et al., 2016) Hur & Gray, 2011 that promotes podocyte injury and death (Raij et al., 2016; Zhou et al., 2017). Importantly, among ROS‐associated pathways linked to Ang II signaling are the canonical transient receptor potential channels TRPC6, and TRPC5, which play a critical role in maintaining podocyte structural and functional integrity (Ma et al., 2016; Yu et al., 2016; Zhou et al., 2017).

Nephrotic urine from patients and rodents contains the zymogen, plasminogen (Plg), and biologically active plasmin, a protease generated from Plg (Raij et al., 2016). Urinary plasmin has been shown to proteolytically activate ENaC, promoting sodium reabsorption and volume expansion that contributes to edema and to hypertension (Ray et al., 2018; Schork et al., 2016; Svenningsen et al., 2009). Studies in human podocytes have shown that plasmin binds to podocyte' membrane receptors and activates Nox2 and Nox4, thereby increasing ROS which degrades synaptopodin (Raij et al., 2016), disrupt the podocyte's cytoskeletal architecture and promotes podocyte death (Raij et al., 2016; Zhou et al., 2017). Here we demonstrated that in hypertensive‐ proteinuric DS‐HS rats urinary Plg‐plasmin is greatly increased by 3‐fold compared to nonhypertensive DS‐LS rats. In addition, we determined that in podocytes Plg‐plasmin reduces the expression of Talin‐1 and CD151, molecules critical for providing structural resistance to barotrauma and maintenance of the integrin‐dependent adhesion of podocytes to the glomerular basement membrane (Pozzi & Zent, 2012; Sachs et al., 2012; Tian et al., 2014; Verheijden et al., 2018) and of the nuclear factor erythroid 2‐related factor 2 (NRF2), a critical transcription factor that regulates the expression of endogenous antioxidant proteins that protect against ROS damage (Hur & Gray, 2011; Nezu et al., 2017). In addition we showed that inhibition of TRPC5 and of TRPC6 (Chung & Shaw, 2017), significantly prevented these potentially injurious effects of plasmin.

Collectively, the novel pathways described above unveil a genetically conditioned interconnection among miR‐155, RAS, TNF‐alpha, and the Wnt‐catenin /alpha‐klotho pathway that is pathophysiologically operative in Dahl rats, a paradigm of salt sensitive hypertension, but not in similarly hypertensive SHR, which are markedly resilient to the renal and vascular consequences of hypertension that lead to CKD/ESRD (Figure 9) (Raij et al., 1984, 1985). These studies also support the notion that once critical proteinuria develops, plasminuria joins in as a “second hit” that promotes further podocyte injury as well as sodium retention volume expansion, and hypertension (Ray et al., 2018; Schork et al., 2016; Svenningsen et al., 2009). We surmise that similar mechanisms might be operative in populations of humans, and therefore, that our findings of the interaction between miR‐155 and alpha‐klotho could be used to identify and/or predict risk of CKD in populations of hypertensive patients and for development of preventive or therapeutic approaches such as administration of recombinant alpha‐klotho as well as, for the evaluation and development of new molecules.

## AUTHOR CONTRIBUTIONS

L.R. and E.A.J. designed the study, L.M.H.B., R.X.T., E.A.J. and L.R. analyzed the data, L.M.H.B. made the figures, and L.M.H.B., L.R. and E.A.J. drafted and revised the paper. All authors approved the final version of the manuscript.

## FUNDING INFORMATION

Financial support for the research was provided by the South Florida VA Foundation for Research and Education, Inc (L.R.) NIH grants RO1DK120660 and P30 CA008748 (E.A.J.) and DOD Grant W81XWH‐21‐1‐0188 (E.A.J.).

## CONFLICT OF INTEREST STATEMENT

None.

## ETHICS STATEMENT

All studies were approved by the Institutional Animal Care Committee at the Miami VA Medical Center.
